# Familial Myelodysplastic/Acute Leukemia Syndromes—Myeloid Neoplasms with Germline Predisposition

**DOI:** 10.3389/fonc.2017.00206

**Published:** 2017-09-12

**Authors:** Renata Lyrio Rafael Baptista, Anna Cláudia Evangelista dos Santos, Luciana Mayumi Gutiyama, Cristiana Solza, Ilana Renault Zalcberg

**Affiliations:** ^1^Departamento de Medicina Interna/Hematologia, Hospital Universitário Pedro Ernesto, Rio de Janeiro, Brazil; ^2^Programa de Genética, Instituto Nacional do Câncer, Rio de Janeiro, Brazil; ^3^Divisão de Laboratórios do Centro de Transplantes de Medula Óssea (CEMO), Instituto Nacional do Câncer, Rio de Janeiro, Brazil

**Keywords:** familial, leukemia, myeloid neoplasms, germline mutations, WHO classification, *GATA2*, *RUNX1*, *CEBPA*

## Abstract

Although most cases of myeloid neoplasms are sporadic, a small subset has been associated with germline mutations. The 2016 revision of the World Health Organization classification included these cases in a myeloid neoplasm group with a predisposing germline mutational background. These patients must have a different management and their families should get genetic counseling. Cases identification and outline of the major known syndromes characteristics will be discussed in this text.

## Introduction

Most cases of myeloid neoplasms are sporadic; however, a small subset has been associated with germline mutations (1–3). The 2016 revision of the World Health Organization (WHO) classification included a group of myeloid neoplasms—such as myelodysplastic syndrome (MDS), MDS/myeloproliferative neoplasms, and acute myeloid leukemia (AML)—with a predisposing, germline mutational background. The presence of underlying genetic alterations or predisposition syndromes is crucial for diagnosing these familial cases (4).

In familial neoplasms, mutations are present in the heterozygous condition, most commonly resulting in loss of functional alleles and subsequent haploinsufficiency, although gain-of-function mutations have also been reported (5). It seems likely, although still unknown, that progression to hematologic malignancy requires the additional acquisition of somatic mutations in bone marrow stem or progenitor cells, probably in the same genes previously affected by germline mutations.

As many genes related to familial predisposition to myeloid neoplasms were also found to be recurrently mutated in sporadic cases, investigation of familial myeloid neoplasms may further provide insights into normal and malignant hematopoiesis and pathogenic mechanisms underlying hematologic malignancies. Moreover, the presence of germline genetic alterations associated to myeloid neoplasms should not be limited to the proband: family members might be at higher risk of developing myeloid neoplasms (6, 7).

Despite efforts to identify familial cases, only a minority with germline mutation can be explained by known genetic factors. The use of next-generation gene sequencing is allowing more cases of the syndrome to be diagnosed, including those without gene mutation (8). However, it is crucial for the germline material availability from the proband and affected family members for this analysis.

Therapy-related MDS/AML seems to be associated with germline mutations in familial cancer predisposition genes. This increases the possibility of these mutations being susceptibility factors for AML development (9, 10).

**Figure 1:**
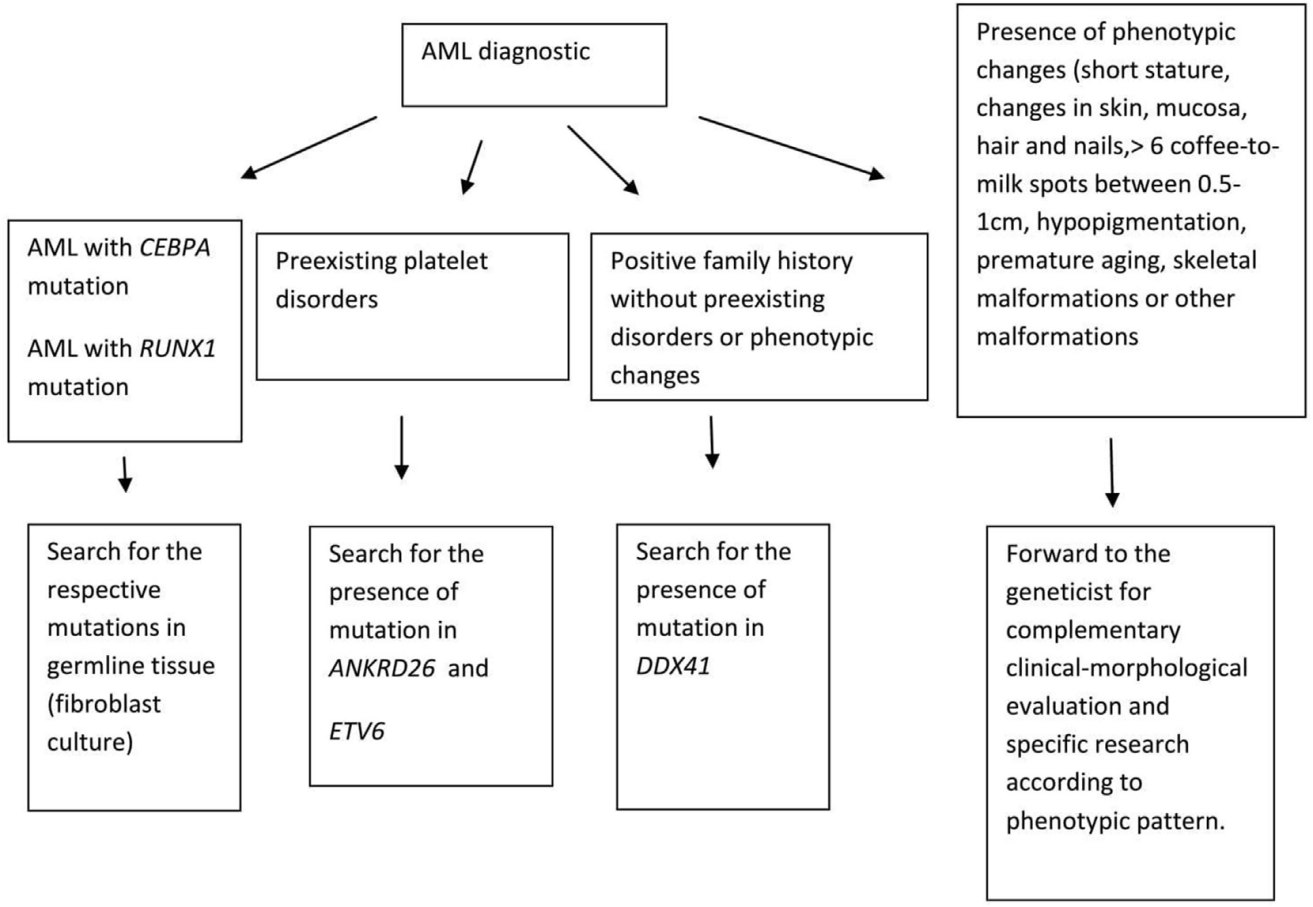
How to identify acute myeloid leukemia (AML) familial cases.

Given the above clinical conditions, physicians should be trained to identify highly suspicious cases of familial neoplasms. A detailed family medical history, collected in all cases of myeloid neoplasms, especially in younger patients, must be mandatory. Close collaboration between hematologists and experienced geneticists in suspected familial cases is crucial. In this review, we will discuss the specific germline alterations associated to familial myeloid malignancies aiming to provide hematologists with diagnosis tools (Figure 1).

## Myeloid Neoplasms with Germline Predisposition

The 2016 revision of the WHO classification included, in a new subset of hematological malignancies associated to germline mutations, the following conditions: (1) myeloid neoplasms with germline predisposition without a preexisting disorder or organ dysfunction, (2) myeloid neoplasms with germline predisposition and preexisting platelet disorders, and (3) myeloid neoplasms with germline predisposition and other organ dysfunction (Table 1) (4).

**Table 1:** Classification of myeloid neoplasms with germline predisposition (4).

**Myeloid neoplasms with germline predisposition without a preexisting disorder or organ dysfunction**
Acute myeloid leukemia with germline CCAAT/enhancer-binding protein-A mutation
Myeloid neoplasm with germline *DDX41* mutation
**Myeloid neoplasms with germline predisposition and preexisting platelet disorders**
Myeloid neoplasms with germline *RUNX1* mutation
Myeloid neoplasms with germline *ANKRD26* mutation
Myeloid neoplasms with germline *ETV6* mutation
**Myeloid neoplasms with germline predisposition and other organ dysfunction**
Myeloid neoplasms with germline *GATA2* mutation
Myeloid neoplasms with germline predisposition with BM failure syndromes
Myeloid neoplasms with germline predisposition with telomere biology disorders
Juvenile myelomonocytic leukemia associated with neurofibromatosis, Noonan syndrome, or Noonan syndrome-like disorders
Myeloid neoplasms associated with Down syndrome

## Myeloid Neoplasms with Germline Predisposition without a Preexisting Disorder or Organ Dysfunction

### AML with Germline CCAAT/Enhancer-Binding Protein-A (*CEBPA*) Mutation

The transcription factor *CEBPA* is allocated in 19q13.1. This gene, consisting of a single exon, is involved in myeloid differentiation. Familial AML with mutated *CEBPA* is an inherited autosomal dominant condition with complete or near-complete penetrance (11, 12). There is not a specific genotype–phenotype presentation. On the other hand, 10–15% of sporadic acute myeloid leukemia with normal karyotype (NK-AML) presents the somatic *CEBPA* mutations. Somatic, bi-allelic *CEBPA* mutations (*CEBPA*dm), found in 10–15% of NK-AML, confer a favorable prognosis, a reason why the identification of *CEBPA* mutation is currently incorporated in routine diagnosis (13, 14). The identification of the germline origin of *CEBPA* mutations in patients with *CEBPA*dm is recommended for discriminating between sporadic and familial cases. Family history is helpful since type or location differences between somatic and germline mutations are presently unknown.

Acute myeloid leukemia diagnosis may be difficult considering the lack of (i) specific clinical features preceding hematological history, (ii) anticipation, and (iii) genotype–phenotype correlation, thus making family history the only source of data for somatic versus familial cases distinction. Identification of familial cases may be impaired by occurrence of *de novo* mutations in a proband or early death of an affected parent without evident clinical AML manifestations. The replacement of the mutated allele in bone marrow can only be achieved by allogeneic stem cell transplantation from a previously tested related donor in whom the mutated allele has been excluded (15).

Finally, the recognition of familial AML with mutated *CEBPA* is essential since penetrance is nearly complete. Genetic counseling is key for managing these cases (16).

### Myeloid Neoplasm with Germline *DDX41* Mutation

DEAD/H-box helicase gene (*DDX41*), allocated in 5q35.3, contains 17 exons and encodes an RNA helicase protein apparently involved in RNA splicing. Its role in hematopoiesis and leukemogenesis remains unknown. Prevalence and penetrance of *DDX41* mutations, as well as prognosis, are unclear (4). However, *DDX41* mutations were present in 1.5% of patients with myeloid neoplasm in a cohort of 1,000 patients. Fifty percent of *DDX41* mutations were germline suggesting that a germline analysis should be considered in these cases (17).

Familial AML with mutated *DDX41* displays a pattern of autosomal dominant inheritance with the characteristics of other MDS/AML syndromes, including a long latency (18). Apart from the family history, there are no preceding clinical signs or symptoms suggesting increased risk for hematologic malignancy.

The majority of familial cases previously identified with this leukemia harbor a heterozygous germline frameshift mutation, *DDX41* c.415_418dupGATG (p.D140Gfs*2), although missense and splice variants have also been described. Another mutation in the other *DDX41* allele occurs in 50% of germline mutation carriers developing MDS or AML, thereby suggesting that *DDX41* is a tumor suppressor gene (17). *DDX41* may play a role in the pathogenesis of myeloid neoplasms with del(5q), since some of these deletions include *DDX4*1 locus, leading to haploinsufficiency. The *DDX41* defects in cases with del(5q) were associated with advanced disease and responsiveness to lenalidomide, a possible therapeutic intervention for otherwise poor-risk disease (19). The overall survival seems to be inferior in *DDX41* mutations or deletions cases and a decreased *DDX41* expression seems to be associated with worse outcomes (14).

Unfortunately, surveillance of unaffected individuals in the general population is not possible. Nevertheless, a bone marrow biopsy with cytogenetic analysis and blood count may be recommended at regular intervals to families with known *DDX41* mutations or deletions and other predisposition syndromes (4).

## Myeloid Neoplasms with Germline Predisposition and Preexisting Platelet Disorders

### Myeloid Neoplasms with Germline *RUNX1* Mutation

Runt-related transcription factor 1 (*RUNX1*) is a protein coding gene allocated in 21q22.12, which contains nine exons. It encodes the DNA-binding subunit of the core-binding factor transcription complex that is essential for normal hematopoiesis (20). Myeloid neoplasms with germline *RUNX1* mutations result from monoallelic *RUNX1* mutations occurring all along this gene, including missense, nonsense, frameshift, insertions, deletions, and, a recently reported, disrupting congenital translocation (21).

Monoallelic *RUNX1* mutations carriers show a heterogeneous range of clinical manifestations: from moderate thrombocytopenia, bleeding, or myeloid neoplasm with frequent strong anticipation, to asymptomatic family members (22).

Management of patients with myeloid neoplasms and germline *RUNX1* mutations depends on clinical presentation. Diagnosis of underlying germline mutations requires allogeneic stem cell transplantation as consolidation therapy provided that *RUNX1* mutations are not carried by related donors (6). Management of asymptomatic *RUNX1* mutation carriers is difficult because guidelines are not presently available for this recently described condition. Considering that myeloid neoplasms with germline *RUNX1* mutations occur with strong anticipation, close follow-ups of the younger members of an affected family are necessary: a baseline blood count with annual checkups, and a bone marrow biopsy in the event of significant changes in peripheral blood counts (7). Finally, as *RUNX1* mutations are found in 32% of sporadic AML (23, 24), translational studies might be relevant for clarifying leukemogenesis in familial platelet disorders (FDP) (23, 24).

### Myeloid Neoplasm with Germline *ANKRD26* Mutation

The Ankyrin repeat domain-containing protein 26 gene (*ANKRD26*), located in 10p12.1, contains 34 exons. Mutations affecting this gene interfere with controlling mechanisms of *ANKRD26* expression, impacting upon megakaryopoieses and platelet production (25). In 9 of 20 unrelated families with autosomal dominant non-syndromic thrombocytopenia-2 (THC2), 6 different mutations were localized in the 5′ promoter region of this gene (26). The overall incidence of these hematologic malignancies was 240/100,000, with acute leukemia accounting for 167/100,000, both above the expected incidences. THC2 has been characterized by platelet dysfunction. The thrombocytopenia is moderate, the mean platelet volume is normal with the presence of aggregation defect, and the bone marrow shows dysmegakaryopoiesis (27, 28). To date, the presence of familial cases of thrombocytopenia and predisposition to myeloid malignancies are the milestones for *ANKRD26* mutations diagnosis (29).

The *RUNX1* or *ANKRD26* mutations should be suspected among patients with thrombocytopenia with a family history of bleeding and/or MDS/AML. If the presence of the mutations is confirmed, patients and family members should be referred to genetic counseling and evaluation of risk of developing myeloid malignancies (7).

### Myeloid Neoplasm with Germline *ETV6* Mutation

The *ETS* variant gene (*ETV6*), located in 12p13.2, contains eight exons and is essential in the development of the embryo and hematopoietic regulation (30). *ETV6* mutations also play a role in hematologic malignancies (31, 32).

The hereditary syndrome of thrombocytopenia with normal platelet size has susceptibility to the development of diverse hematologic malignances. It is transmitted as a dominant trait associated with germline and missense *ETV6* mutations (33). Patients present bleeding and thrombocytopenia and are often misdiagnosed with immune thrombocytopenia (34).

Three hotspot positions were identified in *ETV6* accounting for p.A369G and p.A399C, at the highly conserved ETS, and p.Pro214L, thereby affecting DNA binding. These mutations were found to deter DNA binding, alter subcellular localization, and decrease the repression of transcription in a dominant-negative pattern, thereby impairing hematopoiesis (25). Most recently, five more possibly pathogenic variants were described: p.I358M, p.A377T, p.R396G, p.Y401N, and p.Y401H (35).

The *ETV6* mutations, such as *RUNX1* and *ANKRD26* mutations, should be suspected among patients with thrombocytopenia with a family history of bleeding and/or hematologic malignances (36). If the presence of the mutations is confirmed, the patient and family members should be referred to genetic counseling. However, until now, there are not sufficient data in literature to suggest standardized treatment guidelines for these patients and their families.

## Myeloid Neoplasms with Germline Predisposition Other Organ Dysfunction

### Myeloid Neoplasms with Germline *GATA2* Mutation

GATA-binding protein 2 gene (*GATA2*), located in 3q21.3, contains seven exons and encodes a GATA transcription factor family zinc finger that is essential in normal hematopoietic (37, 38).

In affected families, *GATA2* mutations were transmitted as highly penetrant autosomal dominant traits with early MDS or AML onset. Onset at early age was reported in patients with syndromic presentations (39). Several families with *GATA2* mutations have been described without any distinctive phenotypic or cytogenetic abnormality (2).

Some clinical syndromes, such as Emberger, MonoMAC, congenital neutropenia, and DCML (dendritic cell, monocyte, and lymphocyte deficiency), are associated with germline GATA2 mutations (40). Emberger syndrome is associated with predisposition to MDS/AL and the presence of systemic manifestations such as primary lymphedema confined to lower extremities and genitals, lymphopenia with low CD4/CD8 count ratio, cutaneous warts, and sensorineural deafness. Emberger syndrome also seems to be associated with eight independent *GATA2* variants (41). The MonoMAC syndrome is connected to MDS/AL predisposition and immunologic defects—such as immunodeficiency, monocytopenia, NK cell, B cell, and macrophage deficiencies—that lead to predisposition to atypical infections and pulmonary alveolar proteinosis (42).

Pedigree analysis showed four different *GATA2* mutations: two in familial AML (p.T354M and p.T355del, both in the second zinc finger domains) (21, 43) and two other in *de novo* AML (p.R308P and p.A350N351ins8) (43).

Transformation to MDS/AL rapidly occurs conferring an adverse prognosis, while indication of allogeneic hematopoietic stem cell transplantation appears to be the most adequate treatment (43).

### Myeloid Neoplasms Associated with Bone Marrow Failure Syndromes

Inherited bone marrow failure syndromes (IBMFS) are rare genetic disorders with characteristic hematopoietic dysfunction and ensuing cytopenias, with high risk of transformation to clonal myeloid malignancies (CMMT) including MDS, AML, or isolated clonal cytogenetic abnormalities (44–48). Hematological neoplasms may occur as the initial manifestation of IBMF; approximately 25% of Fanconi anemia patients lack the typical disease phenotype such as short stature and radial ray anomalies (49, 50). Adult and pediatric *de novo* CMMT are not precisely defined, although diagnosis is based on peripheral blood cell counts and types, bone marrow blasts, cellularity, cytogenetic analysis, and dysplasia presence. A widely accepted definition of IBMFS-associated CMMT is, however, not presently available. Diagnosis of pediatric MDS is based on peripheral blood counts, marrow morphologic dysplasia, and blasts (51, 52). These are valuable indicators for defining MDS. Nevertheless, their applicability to IBMFS-associated MDS in the absence of transformation has not been tested. The risk of developing CMMT in patients with IBMFS has been estimated to be 2,284-fold higher than in general population (53).

The differential risk of developing CMMT among patients with various IBMFS types has not been precisely estimated due to dearth population-based data (54). Despite IBMFS types sharing several clinical and morphological phenotypes, IBMFS genes might be involved in different pathways, a reason why mutations in different IBMFS genes might have disparate malignant effects. This provides a rationale for routine leukemia surveillance in children with Fanconi anemia and Shwachman–Diamond syndrome.

### Myeloid Neoplasms Associated with Telomere Biology Disorders

Malignancies associated with telomere biology disorders result from mutations in nine different genes that induce an abnormal maintenance of telomeres leading to chromosome instability and apoptosis (55, 56). Dyskeratosis congenita (DKC), an X-linked recessive disease, is characterized by nail dystrophy, abnormal reticular skin pigmentation, and oral leukoplakia (57). The clinical presentation may vary, resulting in patients with constitutional defects while cancer and MDS predisposition are distinctive. Excessive telomere shortening in Xq28, where the X-linked gene *DKC1* (Dyskerin) is located, leads to genetic instability and high cancer risk (58, 59). A high frequency of hematological malignancies is observed in DKC: approximately 200-fold for AML and 2,500-fold for MDS in relation to the normal population, a reason why the affected patient must be properly screened (60). Deleterious mutations affecting *TERT* (telomerase reverse transcriptase), a gene in 5p15.33, or *TERC* (telomerase RNA component), a gene in 3q26.2, are transmitted as autosomal dominant traits with heterogeneous phenotypes and incomplete penetrance (61). Phenotypes range from normal to severe hematological neoplasms, with variable age at onset and anticipation (62). This fact should not be ignored since children inheriting *TERT/TERC* mutations might present earlier clinical manifestations although their parents carrying the same mutations may not. Clinical presentation may include isolated idiopathic pulmonary fibrosis, hepatic cirrhosis, early-onset anogenital or head and neck cancer, and combinations of these features. The frequency of these associated manifestations is still unknown. These findings point out the need of *TERC* and *TERT* screening in families with more than one case of MDS/AL and/or patients with subtle blood abnormalities, failure to mobilize stem cells or clinical manifestations in other organs or systems (6).

### Juvenile Myelomonocytic Leukemia (JMML) Associated with Neurofibromatosis, Noonan Syndrome, or Noonan Syndrome-Like Disorders

Juvenile myelomonocytic leukemia is an aggressive myelodysplastic/myeloproliferative malignancy. Most JMML cases are associated with somatic gain-of-function mutations in components of the RAS/MAPK signal transduction pathway (63). A minority of cases arise in young children with Noonan syndrome, a genetic disorder with increased RAS/MAPK signaling. Fifty percent of Noonan syndrome patients and 35% of JMML cases carry gain-of-function mutations in *PTPN11* (protein-tyrosine phosphatase, non-receptor-type, 11), altering SHP-2, a tyrosine phosphatase involved in the regulation of the RAS/MAPK pathway (64).

In Noonan syndrome, JMML may occur due to *PTPN11* germline mutations with similar clinical features to children with JMML arising from *PTPN11* somatic mutations, although with a generally better outcome. Mutations in *PTPN11, RAS, NF1*, and *CBL* are exclusive in JMML indicating that one hit in the RAS/MAPK pathway is sufficient for leukemogenesis (65).

#### Neurofibromatosis

Approximately 10–15% of pediatric JMML occur in association with neurofibromatosis type I, disease resulting from mutations in the neurofibromatosis type I gene (*NF1*) that encodes neurofibromin. Neurofibromin is a molecule that regulates several intracellular processes, such as the RAS–MAPK pathway (66). Neurofibromatosis type I is an autosomal dominant disorder with a clinical presentation that includes *café au lait* spots, ocular Lisch nodules, and skin fibromatous tumors. The development of benign and malignant tumors is high in these individuals.

#### Noonan Syndrome-Like Disorders

Germline mutations affecting *CBL* (casitas-B-lineage lymphoma protooncogene), a gene located in 11q23.3, may result in variable Noonan syndrome-like phenotypes (OMIM#613563). In these patients, presence of neurologic features is relatively high, with predisposition to JMML, low prevalence of cardiac abnormalities, reduced growth, and cryptorchidism (67).

Finally, germline mutations affecting *SHOC2* (suppressor of clear, *C. elegans*, homolog), a gene located in 10q25.2, usually result in Noonan syndrome-like phenotypes (OMIM#607721) and JMML, and a classic Noonan syndrome in a small proportion of affected individuals. A recurrent missense *SHOC2* mutation (4A>G) has been identified in a NS subgroup with growth hormone deficiency, hyperactive behavior improving with age, hair anomalies, darkly pigmented skin with eczema or ichthyosis, hypernasal speech, and mitral valve dysplasia and septal defects respective with classic NS (68).

### Myeloid Neoplasms Associated with Down Syndrome

The myeloid neoplasms associated with Down syndrome are Down syndrome transient abnormal myelopoiesis (DS-TAM) and myeloid leukemia Down syndrome (ML-DS). The GATA-binding protein 1 gene (*GATA1*), located in Xp11.23, encodes a zinc finger DNA-binding transcription factor that is critical for the normal development of hematopoietic cells. *GATA1* mutations are a hallmark of DS-TAM and ML-DS (69). All *GATA1* mutations have been allocated to exon 2 (or rarely exon 3) (70). Regardless of mutation types, all of them have been found to generate a premature stop codon, with transcription initiating from an in-frame ATG triplet in codon 84 resulting in a short *GATA1* isoform (~40 kDa), called “GATA1s,” lacking an N-terminal transactivation domain. DS-TAM/ML is associated with a typically constitutional trisomy 21, although some patients have been shown to be mosaics for trisomy 21 or carriers of translocations involving chromosome 21. The lack of a typical DS phenotype cannot, therefore, exclude DS-TAM. DS-TAM is clinically and morphologically undistinguishable from AML, with blasts with morphologic and immunologic characteristics of the megakaryocytic lineage. It is unique to Down syndrome newborns, present in approximately 10% of DS but infrequent in phenotypically normal trisomy 21 mosaics (71).

Down syndrome transient abnormal myelopoiesis shows a heterogeneous clinical presentation and most patients are asymptomatic. This is the reason why it is incidentally diagnosed in peripheral blood checkups showing thrombocytopenia or thrombocytosis, high white blood cell counts, excess of blasts, and, frequently, nucleated red blood cells. Hepatomegaly is a common feature, while infrequent severe manifestations may include fetal hydrops, liver failure, jaundice, coagulation defects, bleeding diathesis, heart failure, pleural effusions, ascites, and/or respiratory failure. Symptoms may appear as early first 3 weeks of life. In most patients, DS-TAM undergoes spontaneous remission within the first 3 months of life (72). ML-DS is frequently preceded by a MDS-like phase that may last for months, characterized by decreasing thrombocytopenia, ineffective erythropoiesis with subsequent anemia, and dysplastic alterations in bone marrow (73).

While coexisting *GATA1* mutations and trisomy 21 might account for DS-TAM, additional alterations in preexisting DS-TAM—*GATA1* seem to be necessary for generating ML-DS. These include trisomy 8 and 21, partial or complete deletions of chromosome 5 and 7, dup(1q), del(16q) (74) and somatic mutations in *JAK1, JAK2, JAK3* (75), *TP53* (76), *FLT3*, and *MPL* in small subsets of cases.

Individuals with DS have a 50-fold increase in the incidence of acute leukemia during the first 5 years of life compared to non-DS individuals. The great majority of DS children with ML-DS are under 5 years of age (77). ML-DS occurs in 20–30% of children with prior history of TAM and leukemia usually occurs 1–3 years after TAM. ML is usually acute megakaryoblastic leukemia in 50% of cases. The clinical course in children with less than 20% blast cells in bone marrow appears to be indolent, with initial presentation of a period of thrombocytopenia. AML prognosis for infants with DS is more favorable than for patients with non-DS AMKL. Treatment with current chemotherapy protocols is associated with 80% rates of event-free survival. Despite the excellent response to therapy, death resulting from toxicity remains a problem, occurring in approximately 7% of cases. Gene expression signatures differ between ML-DS and non-DS AMKL (78). These differences cannot be attributed to the simple presence of an additional set of chromosome 21 genes. Overexpression of *c-Kit, c-MYC*, and *GATA2*, occurs in ML-DS, in contrast to non-DS AMKL cases, indicating that these malignancies are different entities (72).

## Conclusion

Diagnosis of inherited forms of MDS/AML in adults has recently increased. The family history of young adults with MDS/AML should be investigated. Following diagnosis of genetic predisposition syndromes, critical clinical conducts must include therapy, HSCT, cancer surveillance, and genetic counseling.

## Author Contributions

All authors contributed to the conception, drafting of the abstract, and its revision for important intellectual content.

## Conflict of Interest Statement

The authors declare that the research was conducted in the absence of any commercial or financial relationships that could be construed as a potential conflict of interest.
